# MD Simulation of Vector–Receptor Pharmacologic Pairs for Tumor-Specific Drug Delivery: Transfer of Boron Atoms by RGD Peptide to αvβ3 Integrin Receptor

**DOI:** 10.3390/cimb48040411

**Published:** 2026-04-16

**Authors:** Ivan Baigunov, Kholmirzo Kholmurodov, Jaloliddin Gafurzoda, Mirzoaziz Husenzoda, Elena Gribova, Pavel Gladyshev, Dara Slobodova, Raisa Gorshkova, Alexey Lipengolts

**Affiliations:** 1 Department of Chemistry, New Technologies and Materials, Dubna State University, 141980 Dubna, Moscow Region, Russia; vanes1997fev@gmail.com (I.B.); elena_g67@mail.ru (E.G.); pglad@yandex.ru (P.G.); 2 Frank Laboratory of Neutron Physics, Joint Institute for Nuclear Research, 141980 Dubna, Moscow Region, Russia; 3 Department of Fundamental Nuclear Interactions, Faculty of Physics, Lomonosov Moscow State University, 119991 Moscow, Russia; 4 S.U. Umarov Physical-Technical Institute (PhTI), Aini Ave. 299/1, Dushanbe 734063, Tajikistan; 5 Department of Technical Operation of Air Transport, Faculty of Transport and Road Infrastructure, Tajik Technical University, Academicians Radjabov Str. 10, Dushanbe 734042, Tajikistan; maximus_509716@mail.ru (J.G.); mirzoazizkhusenov@gmail.com (M.H.); 6 MEZON LLC, 141983 Dubna, Moscow Region, Russiagorshkova.raisa@gmail.com (R.G.); 7 N.N. Blokhin National Medical Research Center of Oncology, Ministry of Health of the Russian Federation, Kashirskoe Shosse, 24, 115478 Moscow, Russia; lipengolts@mail.ru

**Keywords:** molecular dynamics, vector–receptor, RGD peptide, αvβ3 integrin, boron

## Abstract

We utilized molecular dynamics (MD) simulations to explore the interaction of the RGD peptide with the αvβ3 integrin receptor, a key process for targeted drug delivery to tumors. The goal of these simulations was to model the transport of boron atoms by the RGD peptide and to characterize the binding event between this vector and its receptor. The study focused on the interaction processes and spatial arrangements of the solvated RGD–integrin system. Simulations were run for 100 ns to achieve relaxed-state configurations. Our model featured two RGD peptides: one pre-localized within the integrin’s binding site and another initially positioned externally. The external peptide was observed to diffuse freely and subsequently bind to the αvβ3 integrin. This spontaneous binding event provides valuable insights into the pharmacological specificity and mechanisms of the RGD–integrin interaction, informing the design of effective drug delivery systems.

## 1. Introduction

This research employed computational molecular dynamics (MD) simulations to investigate the interaction between a “vector–receptor” pharmacological pair. The primary goal was to model potential mechanisms and processes for targeted drug delivery to tumors. Specifically, these MD calculations aimed to simulate the transport of boron atoms via the RGD peptide, facilitating subsequent interactions with the pharmacological vector–receptor pair. The chosen pair was RGD and integrin αvβ3. Here, RGD, a peptide comprising L-arginine, glycine, and L-aspartic acid, serves as the vector. The receptor is integrin αvβ3, a vitronectin receptor and a known marker for tumor neoangiogenesis, composed of integrin alpha V and integrin beta 3 subunits. It is important to note that RGD peptides can bind to individual subunits or both simultaneously [1,2,3,4,5,6]. The study proposes to assess the binding of the RGD peptide to αvβ3 integrin through molecular calculations, considering both single and multiparticle (cluster) delivery of boron atoms to the receptor. These simulated processes are expected to illuminate potential pathways for the specific delivery of boron to tumor sites.

This research is dedicated to the examination of promising and, in our assessment, the most feasible mechanisms for the specific binding and delivery of molecular reagents to tumors through a “vector–receptor” framework [1,2,3,4,5,6,7,8,9]. The study also encompasses a description of the known amino acid transport mechanism via the LAT1 receptor, a system integral to boron neutron capture therapy (BNCT) [2,6,7,8,9]. It is worth noting that the role of boron in BNCT is characterized through the selective destruction of malignant tumor cells by accumulating a stable boron-10 isotope in them and subsequent irradiation with epithermal neutrons. The BNCT (boron neutron capture therapy) is a promising method of treating malignant tumors, and this method involves the targeted destruction of cancer cells without surgical intervention. In the BNCT method, the key importance is not the “take-off length” of the neutrons, but rather the depth of their penetration into tissues until the boron-10 capture. BNCT is based on the epithermal neutrons (energies from 0.5 eV to 10–20 keV) and penetration depth of 8–10 cm. The epithermal neutrons, slowing down to thermal energies, are able to penetrate body tissues to a depth of 8–10 cm, which makes the deep-lying tumors treatment possible, such as glioblastomas of the brain, while thermal neutrons are absorbed by the skin. Regarding the mileage of secondary particles, after neutron capture by the boron-10 nucleus, a nuclear reaction occurs, and the resulting alpha particle and lithium-7 nucleus have a low free path of only 5–9 μm (approximately the diameter of one cell). This ensures the selective destruction of only the cancer cell containing boron; thus, neutrons travel centimeters in tissues, but the destructive reaction occurs at the micrometer level [2,3,4,5,6,7,8,9].

Given the inherent challenges in the experimental validation of these phenomena, this work leverages computational molecular dynamics (MD) to simulate and analyze the interactions of pharmacological vector–receptor pairs. MD simulations represent a premier class of methods for modeling the specific action and interaction of drugs with receptors. Consequently, the central aim of this paper is the elucidation of the dynamic and structural properties of the relevant peptide–protein complexes.

For practical applications, two primary strategies are suggested for directing boron-containing nanoparticles to their targets:

1. utilizing a peptide with the RGD sequence, known for its specific affinity for integrin αvβ3 (a receptor frequently overexpressed in tumors);
2. employing folic acid, which targets the FR-α receptor, also commonly found in abundance on tumor cells.

The literature data for the RGD peptide vector system in specific delivery to the avb3 integrin receptor and description of the results and examples of targeted drug delivery through this receptor, as well as the molecular basis for the targeted binding of RGD-containing peptide to integrin αvβ3 and novel linear peptides with high affinity to αvβ3 integrin in precise tumor identification, can be found in refs. [2,7,9,10,11].

Thus, the goal of these computational MD calculations and virtual experiments is to model the interaction processes of the pharmacological pair “vector–receptor” (Table 1):

The molecule αvβ3, identified as an integrin and the vitronectin receptor, serves as an indicator of new blood vessel formation in tumors. This integrin is composed of two distinct subunits: integrin alpha V and integrin beta 3. It is important to recognize that RGD peptides possess the capability to attach to either of these subunits individually, or to both at the same time. At present, it remains unclear if RGD peptides exhibit varying degrees of affinity for αvβ3 integrins found in mice compared to those in humans. Therefore, the plan is to quantify the binding interaction between RGD and both murine and human αvβ3 integrins. This principle extends to other potential strategies for targeted delivery of boron to tumor sites. Furthermore, for comparative purposes, it is also suggested to assess the specificity of the sole established method for boron delivery, which relies on amino acid transport through LAT1 pathways (Table 2).

**Figure 1:**
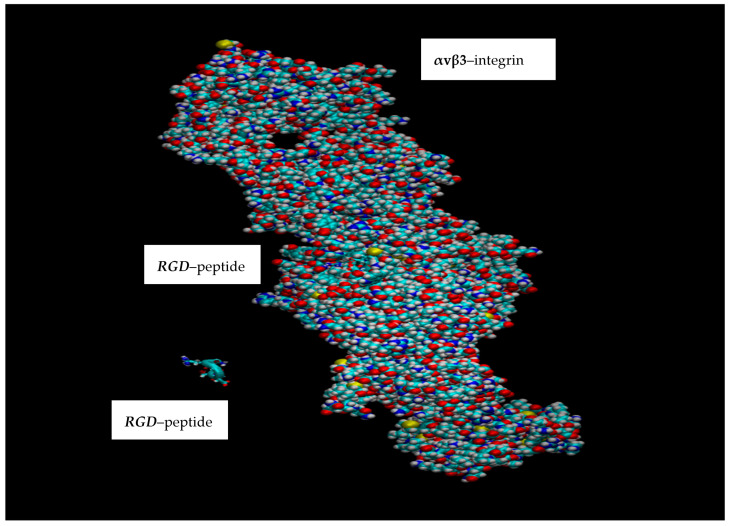
Overview of the RGD–integrin αvβ3 interaction. The αvβ3 integrin receptor is on the right, and small peptides with the RGD amino acid sequence (L-arginine, glycine, L-aspartic acid) are on the left.

**Figure 2:**
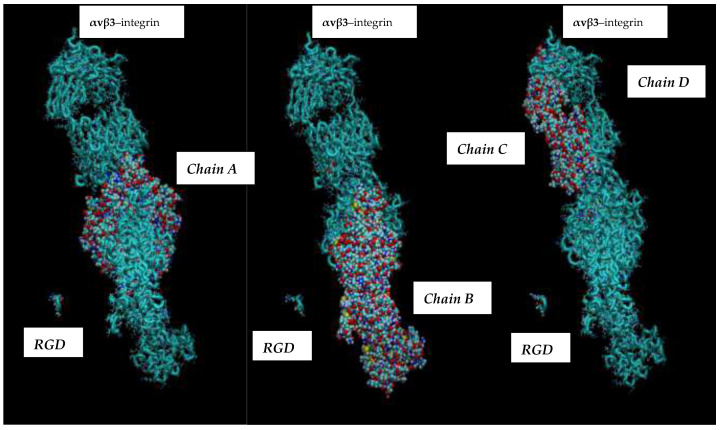
A general representation of the RGD–integrin αvβ3 pairing. The integrin, identified as the αvβ3 receptor, is positioned on the right, and small peptides containing the RGD amino acid sequence (L-arginine, glycine, L-aspartic acid) are on the left.

Molecular dynamics (MD) simulations provide a powerful lens for investigating and comprehending atomic and molecular behaviors across a broad spectrum of physical and biochemical contexts. Their application is extensive, spanning physics, chemistry, and the life sciences, and they are instrumental in the computer-assisted development of novel materials and pharmaceuticals [12,13,14,15,16,17,18]. Presently, virtual MD experiments are being conducted using the PDB (Protein Data Bank) entry 3ZE2, which depicts the complex of the integrin αvβ3 headpiece with an RGD peptide. This molecular assembly comprises multiple polypeptide chains (e.g., MOL_ID: 1, CHAIN: A, C, spanning residues 32–488; MOL_ID: 2, CHAIN: B, D, covering residues 27–498) and includes peptides such as the RGD peptide (MOL_ID: 5, CHAIN: I, J), originating from species like *Homo sapiens* and *Chinese hamster* (see Figure 1 and Figure 2) [16].

## 2. Materials and Methods

This section details the core parameters and computational methods employed in our molecular dynamics (MD) simulations. For these simulations, we leveraged both central processing units (CPUs) and graphics processing units (GPUs) within the AMBER software suite. Specifically, our computational setup included a 16-core cluster and GeForce GPUs (GTX 1080Ti) for running MD simulations with AMBER 18 (utilizing the pmemd.cuda module for GPU acceleration). We have employed the Amber 2018 program code and its reference manual for guidance [12,13,14]. The primary computations were conducted on the servers of the “HybriLIT” Heterogeneous Platform, part of the Multifunctional Information and Computing Complex (MICC) at the M.G. Meshcheryakov Information Technologies Laboratory, Joint Institute for Nuclear Research (JINR, Dubna). This platform is a dual-system architecture comprising the Govorun supercomputer and the HybridIT training and testing facility, featuring a CPU component built on advanced Intel architectures (Intel Xeon Phi and Intel Skylake) and a GPU component based on NVIDIA DGX-1 Volta. Additional calculations were performed on a local server at the Frank Laboratory of Neutron Physics (JINR), equipped with two 4-core 64-bit Intel Xeon E5-2640 processors running at 2.4 GHz, 8 GB of RAM, and the Linux CentOS 8 operating system. This local server also houses an NVIDIA GP104 [GeForce GTX 1070] GPU, alongside Intel Xeon E7 v4/Xeon E5 v4/Xeon E3 v4/Xeon processors. The principal production MD simulations (applicable to both CPU and GPU execution, and common to many simulation types) were carried out for the PDB entry 3ze2 (DOI: https://doi.org/10.2210/pdb3ZE2/pdb) [16,17].

**Figure 3:**
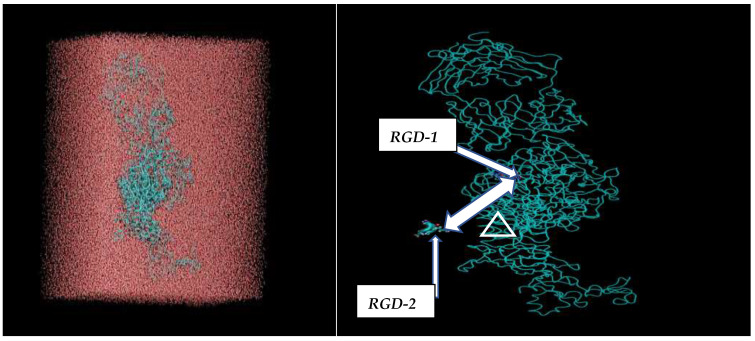
Initial system, RGD peptides + αvβ3 integrin receptor solvated with water (TIP3P model) for subsequent MD calculations.

**Figure 4:**
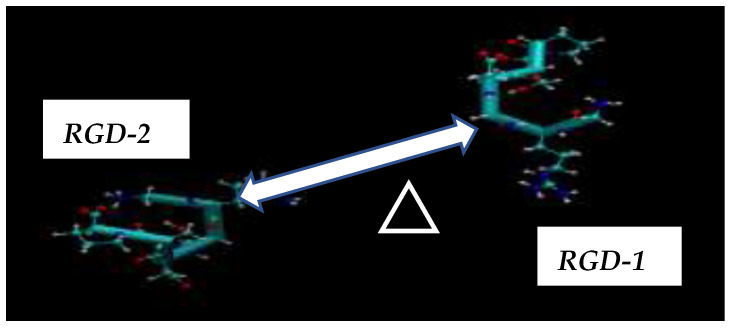
Two RGD peptides are located outside (left: RGD-2) and inside (right: RGD-1) of the αvβ3 integrin receptor, with the distance between them denoted as Δ [RGD-1 (GLY, PRO)–RGD-2 (GLY, PRO)]. The RGD peptides consist of a sequence of amino acids (L-arginine, glycine, L-aspartic acid).

Using the Amber 18 code (CPU/GPU), molecular dynamics (MD) simulations were performed on a system containing the RGD peptide, the αvβ3 integrin receptor, and TIP3P water (see Figure 3 and Figure 4). The molecular visualization and analysis of the MD trajectories were done using the VMD (Visual Molecular Dynamics; ver 1.9.4). It is worth noting that VMD is a molecular visualization and analysis program designed for biological systems such as proteins, nucleic acids, lipids, etc., and is a super handy program in generating high-quality images and animations [16]. The simulation workflow involved three initial stages: energy minimization, NVT relaxation, and NPT relaxation, collectively lasting 10 ns for dynamic equilibration. The Amber GPU code was employed for performance, with unlinked list cells not being recomputed. Once the system reached equilibrium, minimal box size fluctuations were anticipated, ensuring smooth production calculations. Periodic boundary conditions and pressure control were applied to symmetrically confine the RGD + αvβ3 integrin + water system, effectively modeling it as a large, thermostatted cubic box (Table S1). MD simulations, utilizing the SHAKE algorithm for hydrogen bonds (without strength evaluation) and a Langevin thermostat, were conducted at 303 K. Thus, we have completed several MD runs for each MD model (see Supplementary Materials) for fifty million time steps (nstlim = 50,000,000, dt = 0.002, cut = 9.0, ntt = 1) at room temperature (temp0 = 303). Constant pressure, anisotropic pressure coupling, and a 10 Å cutoff for non-bonded interactions were implemented. The Amber18 CPU/GPU-accelerated simulations (pmemd/pmemd.cuda) followed three core setup steps for the main production MD models:

1. Minimizing the system to eliminate steric clashes.
2. Incrementally raising the system’s temperature to the target.
3. Allowing the system to equilibrate at the target temperature.

The initial phase of our study involved system optimization through the application of constraints on the backbone and specific other atoms. Default charge distributions from tleap’s jumprc.constph and jumprc.conste states were used for titratable residues. Sander was chosen over pmemd for minimization to closely monitor energy changes. Subsequently, constant-volume heating simulations were performed on the minimized structures, gradually increasing the temperature from 10 K to a target of 303 K over 2.0 ns. Backbone atom constraints were kept, but with a lower force constant. These heating simulations were executed using Sander or Sander.MPI, and pmemd or pmemd.MPI, excluding the GPU-accelerated pmemd.cuda. The next step focused on equilibrating the complete system (RGD peptides, αvβ3 integrin receptor, and water) at 303 K. Following this, explicit solvent modeling was implemented under constant pressure to ensure system density stabilization. The simulations spanned 50–100 ns, incorporating explicit solvent representations.

**Figure 5:**
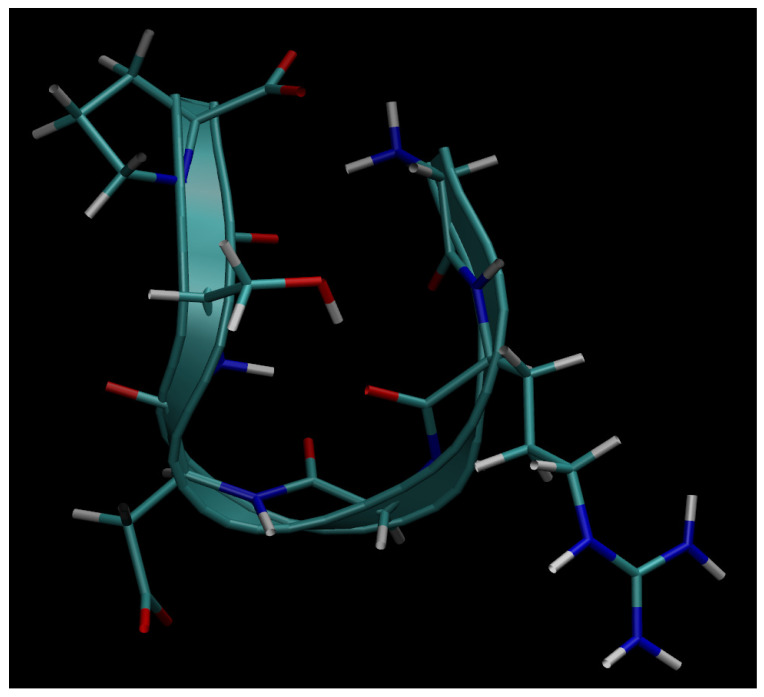
A structural configuration of the RGD peptide being to interacting with and inserting into the receptor integrin αvβ3. The small RGD peptide containing the specific amino acid residues (L-arginine, glycine, L-aspartic acid, etc.).

For the numerical MD simulation implemented in this study, the multifunctional AMBER-18 package with a fast implementation of the “pmemd.cuda” module on a CPU/GPU cluster machine is used. The numerical experiment significantly accelerates the performance of the AMBER-18 computer code (“pmemd.cuda”) and is aimed at the specificity or binding of the RGD peptide system (a detailed structure is shown in Figure 5) to the αvβ3 integrin receptor.

**Figure 6:**
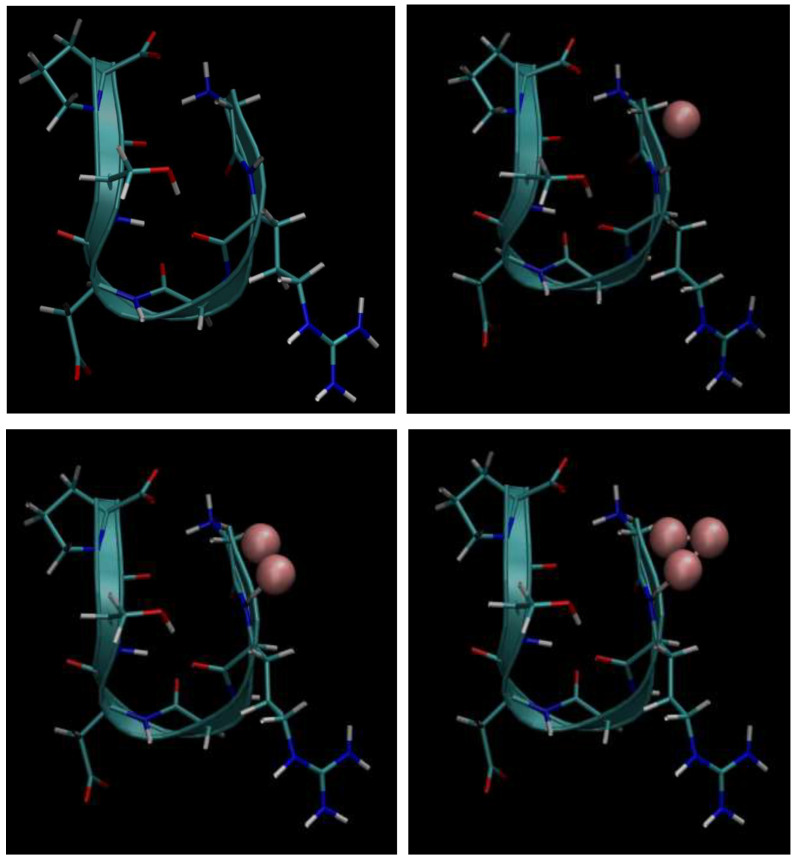
Binding of the boron atom in single and multiparticle forms to the amino acid sequence of the RGD peptide vector for subsequent specific delivery to the αvβ3 integrin receptor.

**Figure 7:**
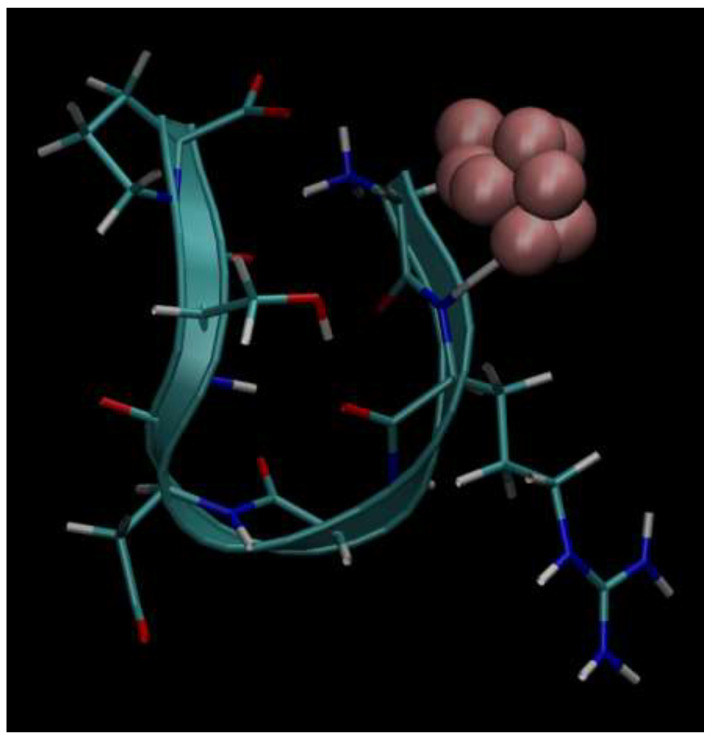
Binding of boron atoms in cluster form to the amino acid sequence of the RGD peptide vector for subsequent specific delivery to the αvβ3 integrin receptor.

**Figure 8:**
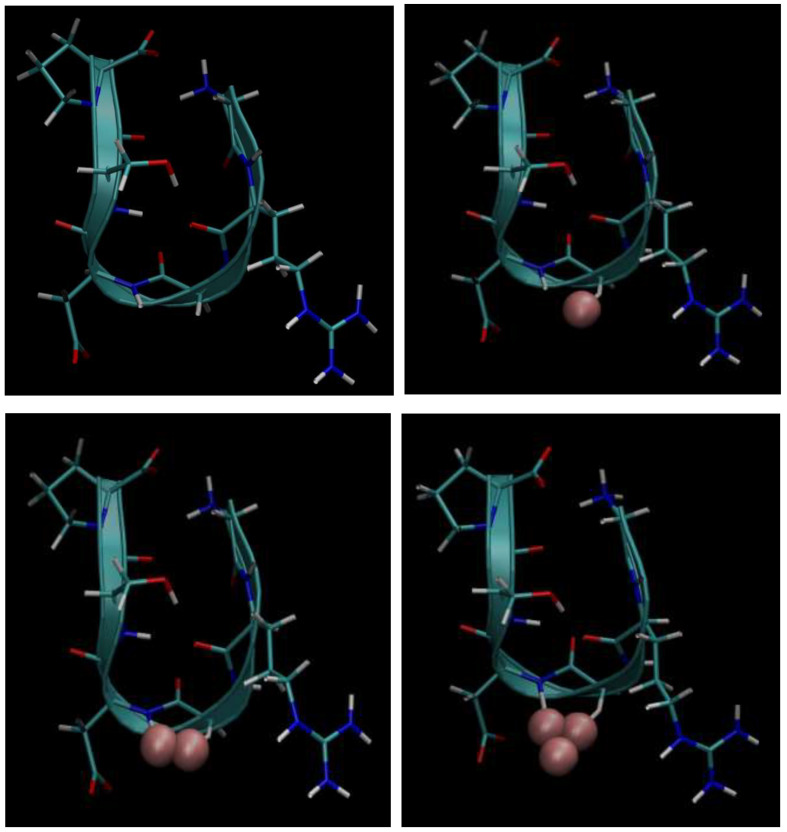
Binding of the boron atom in single and multiparticle forms to the amino acid sequence of the RGD peptide vector for subsequent specific delivery to the αvβ3 integrin receptor.

**Figure 9:**
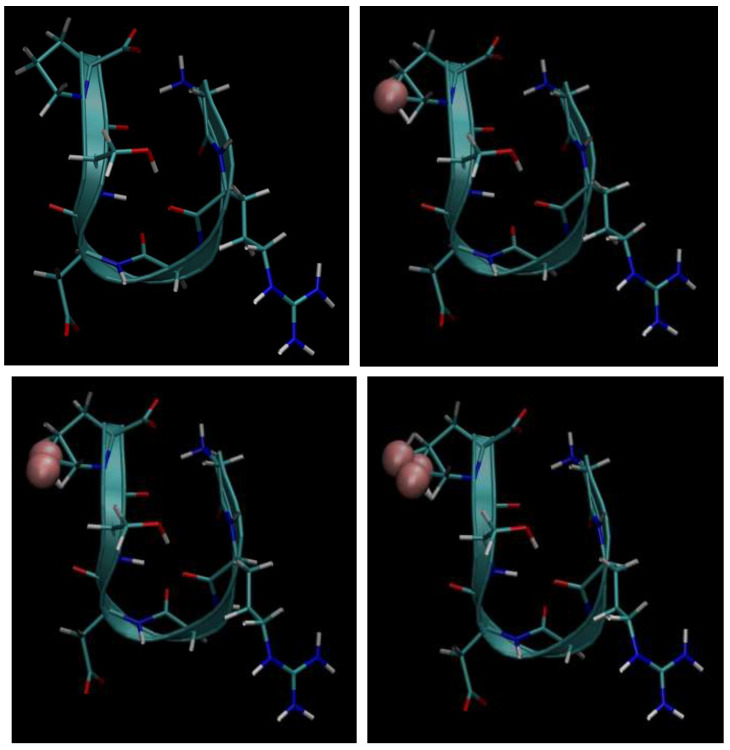
Binding of the boron atom in single and multiparticle forms to the amino acid sequence of the RGD peptide vector for subsequent specific delivery to the αvβ3 integrin receptor.

**Figure 10:**
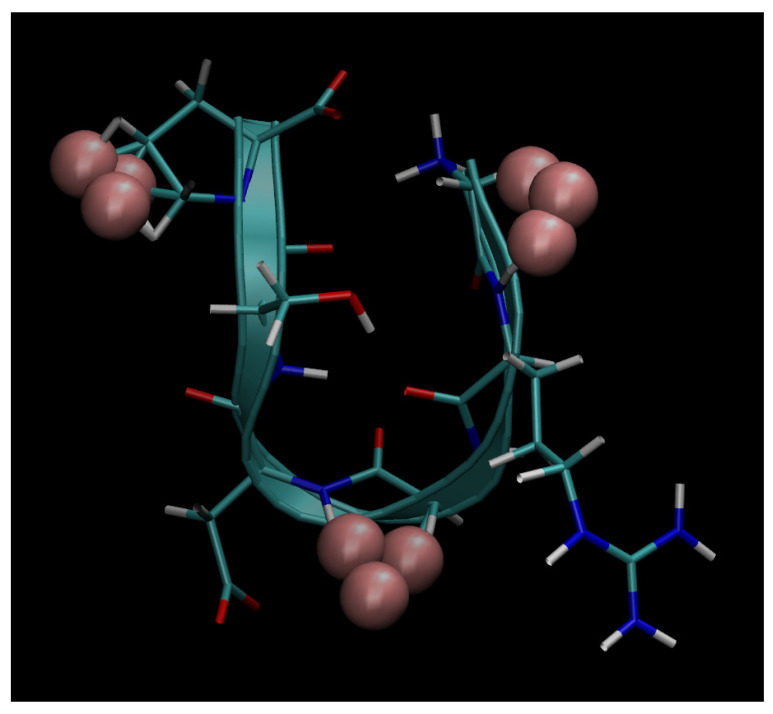
Binding of boron atoms in cluster form to the amino acid sequence of the RGD peptide vector for subsequent specific delivery to the αvβ3 integrin receptor.

Next, below in Figure 6, Figure 7, Figure 8, Figure 9 and Figure 10, we present the multiple molecular configurations of the RGD peptides and αvβ3 integrin receptor to evaluate the possible binding mechanism of the RGD to αvβ3 integrin during single-particle or multiparticle (cluster) delivery of boron atoms to the receptor. The MD results may shed light on possible pathways for specific boron delivery to tumors. The RGD peptide begins and ends with (GLY, PRO), amino acids that contribute hydrophobicity and lack charge in their side chains. The presence of (ARG) at the peptide’s termini signifies a positively charged side chain in neutral aqueous solutions, stemming from an isoelectric point significantly above 7.0, leading to a net positive charge under these conditions. Proteins dominated by such amino acids are designated as alkaline, reflecting their isoelectric points also exceeding 7.0. In contrast, (ASP) carries a negative charge on its side chain, with an isoelectric point below 7.0. Finally, SER is an amino acid with a hydrophilic, uncharged side chain, which confers the ability for protein regions containing it to undergo hydration and engage in hydrogen bonding with other similar residues. The generated multiple MD configurations in Figure 6, Figure 7, Figure 8, Figure 9 and Figure 10 thereby demonstrate all key binding sites of the boron atoms to the RGD peptide in a specific delivery to the αvβ3 integrin receptor.

## 3. Results and Discussion

**Figure 11:**
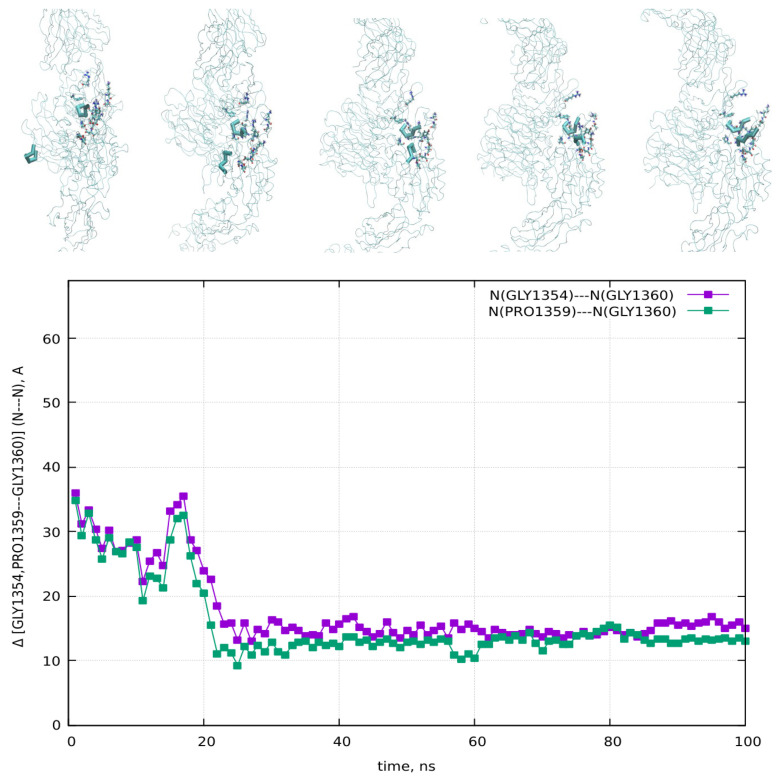

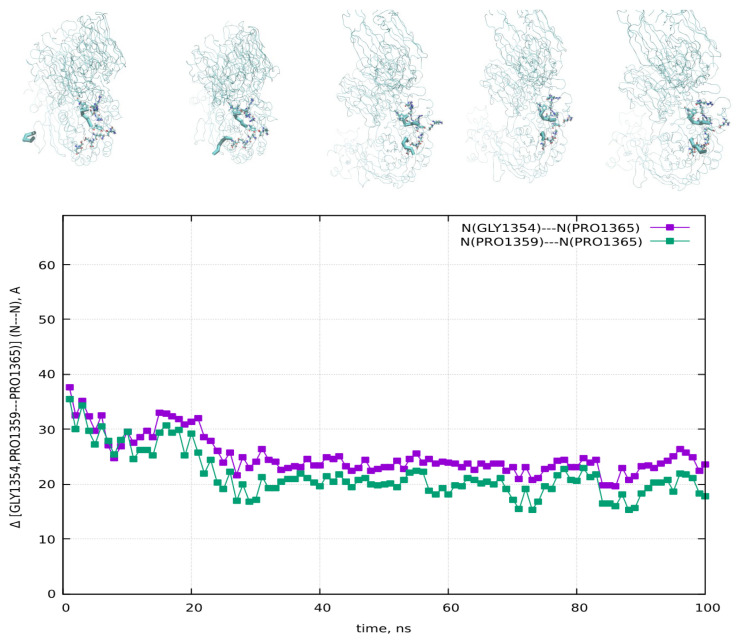
(**top** and **bottom**) Visualization of the RGD-1 and RGD-2 peptide vectors interacting with the αvβ3 integrin receptor and their orientation in relaxed configurations at the start (t = 0), during intermediate stages, and at the end of the simulation (t = 100 ns), along with the dynamic variations in the distances between RGD-1 (GLY, PRO) and RGD-2 (GLY, PRO) are shown.

The molecular dynamics (MD) simulations, guided by the schematics in Figure 3 and Figure 4, allowed for the computation of all potential distances between the RGD-1 (GLY, PRO) and RGD-2 (GLY, PRO) peptides. These MD findings, visualized as distance plots in Figure 11 (both top and bottom panels), reveal a diminished spatial alignment of the RGD-1 and RGD-2 peptide vectors within the αvβ3 integrin receptor. Specifically, the RGD-2 peptide, initially external to the receptor, gradually inserts and aligns nearly parallel to the RGD-1 peptide over extended 100 ns simulations, characterized by dynamic shifts and free diffusion. The distance distribution diagrams in Figure 11 suggest a tight integration of the external RGD-2 peptide alongside the RGD-1 peptide, which is already embedded within the αvβ3 integrin receptor.

**Figure 12:**
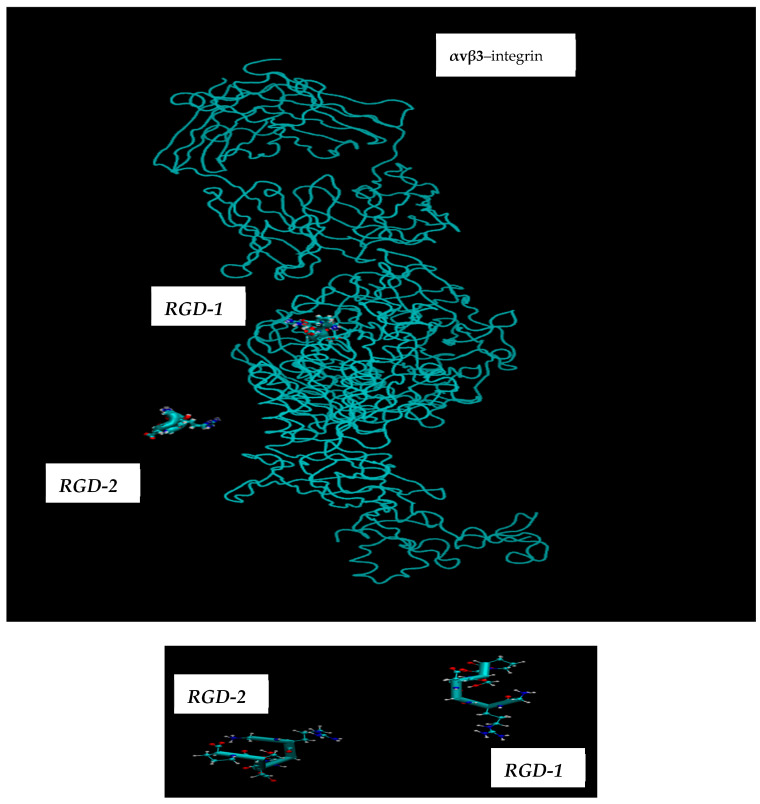
(**top** and **bottom**) Two RGD peptides located outside (RGD-2) and inside (RGD-1) the αvβ3 integrin receptor are shown.

The RGD peptide, consisting of a sequence of amino acids (L-arginine, glycine, L-aspartic acid), is one of the important agents that regulate the mechanisms of delivery and specific binding for selected ions. It is similar to a small drug or related reagents delivery to the tumor in the “vector–receptor” format that could be used in the boron neutron capture therapy (BNCT) [2,11,15,16,17,18,19,20,21,22,23,24]. It should be noted that this sequence (RGD peptide) is capable of specifically binding to the αvβ3 integrin overexpressed in the tumor node. The mechanism of amino acid transport through the LAT1 receptor was studied in [9,10,11]. Below we present the results of molecular dynamics modeling of the possible boron atom delivery by the RGD peptide containing the amino acids (L-arginine, glycine, L-aspartic acid, etc.) in its different location sites (see Figure 12: top and bottom). Molecular dynamics simulation results illustrate the possible interactions of pharmacological vector–receptor pairs with the dynamical features of the boron delivery in single and cluster implementations. Thus, we simulate the two RGD peptides located outside and inside the αvβ3 integrin receptor and the subsequent effects of the interaction of peptide–protein complexes, as in Figure 12 (top and bottom).

**Figure 13:**
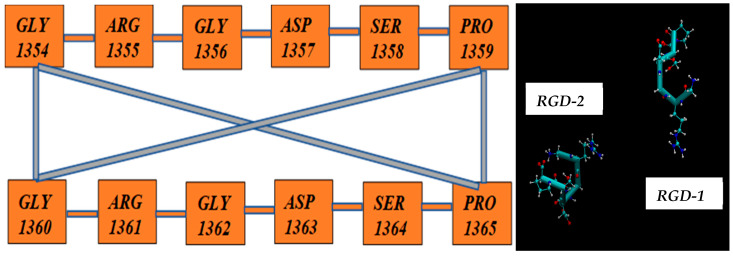
(**left** and **right**) Amino acid sequence of peptide vectors RGD-1,2 with all possible distributions of distances between them, Δ [RGD-1 (GLY, PRO)–RGD-2 (GLY, PRO)].

The aim of the presented MD computations was to model the transport of boron atoms by the RGD peptide in subsequent interaction processes of the pharmacological vector–receptor pair: the RGD-αvβ3 integrin pair, where the vector is RGD (a peptide containing the amino acid sequence L-arginine, glycine, and L-aspartic acid) and the receptor is αvβ3 integrin. It should be noted that RGD peptides are capable of binding to each of the subunits individually or to both simultaneously. To observe the natural contacts and binding of RGD peptide to αvβ3 integrin, we have generated and long-calculated multiple molecular configurations (several dozen MD models each 50–100 ns). One of the RGD peptide (RGD-1) was embedded within the αvβ3 integrin (which is a vitronectin receptor as a marker of tumor neoangiogenesis and containing of two parts: integrin alpha V and integrin beta 3); another RGD peptide (RGD-2) was allowed to freely diffuse from outside of the αvβ3 integrin receptor, so its configuration shape changes arbitrarily throughout the entire model cell. Moreover, one of the two RGD peptides (RGD-1) is a peptide as in the original PDB file and located within the αvβ3 integrin receptor; its dynamics remain localized throughout the 50–100 million steps of the structural dynamical changes and integration of the equations of motion (Figure 13: left and right).

Let us note some design features of RGD peptides, i.e., “Pharmacological” feasibility of choosing a peptide structure for the purpose of their effective binding to the αvβ3 integrin receptor:

**RGD-1** peptide (residue 1354–residue 1359):

**––GLY1354–ARG1355–GLY1356–ASP1357–SER1358–PRO1359––**

**RGD-2** peptide (residue 1360--residue 1365):

**––GLY1360–ARG1361–GLY1362–ASP1363–SER1364–PRO1365––**

Thus, presented above in Figure 6, Figure 7, Figure 8, Figure 9, Figure 10, Figure 11, Figure 12 and Figure 13, MD simulated multiple molecular configurations of the RGD peptides and αvβ3 integrin receptor illustrate and evaluate some possible binding mechanisms of the RGD-2 to αvβ3 integrin receptor + RGD-1 during a single-particle or multiparticle (cluster) boron atom delivery to the receptor. The MD results may shed light on possible delivery routes for specific boron(s) to tumor(s). In particular, the computer-designed RGD-2-peptide + boron(s) and receptor + RGD-1-peptide that MD modeled above fully correspond to their structural behavior in vivo. The (GLY, PRO) amino acids contribute hydrophobicity and lack of charge in their side chains; the (ARG) at the peptide’s termini signifies a positively charged side chain in neutral aqueous solutions, stemming from an isoelectric point significantly above 7.0, leading to a net positive charge under these conditions (it is worth noting that proteins dominated by such amino acids are designated as alkaline, reflecting their isoelectric points also exceeding 7.0); the (ASP), in contrast, carries a negative charge on its side chain, with an isoelectric point below 7.0; and finally, the (SER) is an amino acid with a hydrophilic, uncharged side chain, which confers the ability for protein regions containing it to undergo hydration and engage in hydrogen bonding with other similar residues. In principle, the MD analysis offers atomic–molecular insights into the RGD peptide’s localization and conformation within the αvβ3 integrin receptor’s binding site. This is particularly relevant given the widespread interest in understanding peptide–protein, protein–protein, and membrane–receptor/enzyme interactions in modern life sciences. Such studies often necessitate considering chemical interactions with solvent molecules and specific surface fragments. Generally, these investigations are crucial for applications in nanobiotechnology and biochemistry, among other fields, and involve a complex interplay of hydrogen, hydrophobic, ionic, and electrostatic forces.

**Figure 14:**
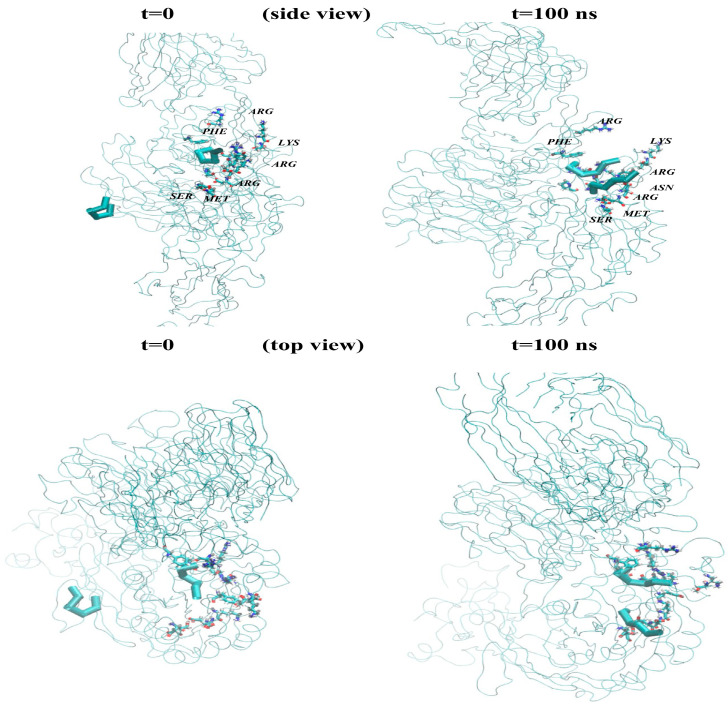
Examining the precise attachment and positioning of the RGD-1 and RGD-2 peptide vectors relative to the αvβ3 integrin receptor’s architecture, depicted from side and top views at the beginning (t = 0) and end (t = 100 ns) of the relaxation process.

**Figure 15:**
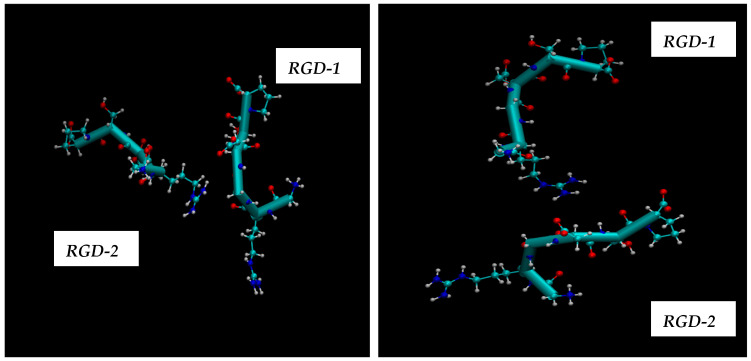
Two RGD peptides with arbitrarily configured dynamics (RGD-2 located outside the αvβ3 integrin receptor) and embedded configuration dynamics (RGD-1 located inside the αvβ3 integrin receptor).

Subsequently, Figure 14 and Figure 15 present the comparative molecular arrangements of the RGD-1 and RGD-2 peptide vectors within the αvβ3 integrin receptor. These illustrations depict the initial (t = 0) and final (t = 100 ns) relaxed states, offering both side and top perspectives. Collectively, Figure 14 and Figure 15 elucidate the distinct binding characteristics and orientations adopted by the RGD-1 and RGD-2 peptide vectors within the αvβ3 integrin receptor’s three-dimensional framework.

The extensive 100-nanosecond molecular dynamics simulations performed above have revealed the specific arrangements and locations of the RGD-2 peptide and the αvβ3 integrin receptor + RGD-1 in a water environment (modeled by using TIP3P). By comparing different configurations, we pinpointed the spatial positions of two RGD peptides: one situated within the αvβ3 integrin receptor (RGD-1, derived from the initial PDB data) and another that freely moved outside the receptor across the simulation cell (RGD-2). Visualizations of these configurations and the distances between the RGD peptides and the αvβ3 integrin illustrate the atomic-level details of natural contact and specific binding. The RGD peptide, characterized by its arginine, glycine, and aspartic acid sequence, acts as a carrier, while the αvβ3 integrin serves as the target receptor. This integrin, a vitronectin receptor associated with tumor blood vessel formation, can bind to RGD peptides either on one of its subunits or on both concurrently. The outcomes of these MD simulations suggest a potential pathway for delivering boron atoms via the RGD peptide for targeted interactions within the RGD peptide–αvβ3 integrin receptor pharmacological system [6,7,8,9,16,17,18,19,20,21,22,23,24].

We have added several animated files in Supplementary Materials where the MD results illustrate the accumulation of RGD-2 peptide in a specific integrin region. These results suggest the existence of multiple binding sites and mechanisms of interaction between the vector and the receptor. However, the MD results clearly demonstrate one of the most likely binding mechanisms between RGD-2 and the receptor surface, regardless of whether the αvβ3 integrin receptor with or without RGD-1 was used. In the case of an integrin that does not contain RGD-1, the RGD-2 + boron(s) would definitely find much more room for incorporation into the receptor. Of the two RGD peptides that were modeled, RGD-1 remains embedded in the protein pocket during the entire simulation period of 50–100 nanoseconds, and the Figures and Animations illustrate the specific binding of RGD-2 in a natural way, since the binding residues of αvβ3 integrin to RGD-1 completely coincide with the initial experimental data, the X-ray measured PDB structure. During multiple (perhaps dozens) MD simulations for RGD-2-peptide + boron atoms, it was necessary to ensure all natural vibrational and diffusion movements, but to preserve the position of boron atoms inside RGD-2-peptide. Thus, we have preserved all the relative positions of the boron atoms bound to RGD-2-peptide during the entire dynamical and diffusional processes. So far, the relative arrangement of boron atoms bound to RGD peptide has not been violated, since the main focus has been on the delivery of RGD-2 + boron(s) to the integrin + RGD-1 receptor. In particular, the FF (force field) used for boron atoms was approximated as partial charges inside boron containing a ligand (RGD-2 + boron), thereby disabling and modifying the prediction of the atom type and bond type, even if the initial FF parameters for the boron atom were not recognized in the running antechamber and related modules. It is worth noting that the dynamic and conformational changes in RGD-2 in the simulated MD cells occur completely naturally, but not every model can lead to the binding of the RGD-2+ receptor. Obviously, there are enough other location sites on the receptor surface where RGD-2 is preferably to be inserted. Statistically, it is possible that there is or may be more than one binding mechanism, but not all of the dozens of MD simulation models ultimately showed favorable binding of RGD-2 + boron(s) and the receptor + RGD-1. Initially, the observation turned out to be unpredictable, and a stochastic event was captured in multiple MD searches (see animated Movies Anim S1 and S2).

Thus, multiple MD calculations of the distance between the vector (RGD-2-peptide + boron atoms) and the receptor (αvβ3 integrin) clearly demonstrate that the entire fragment of RGD-2 + boron(s) is inserted into the receptor naturally. It is worth noting that we aimed at binding the entire RGD-2 + boron fragment(s), rather than specifically binding selected residues and the receptor. The mechanism of dynamical interactions and diffusional movements between the RGD-2-peptide carrier and the αvβ3 integrin receptor is illustrated with an accuracy of millions of time steps and multi-configuration analysis.

## 4. Conclusions

This study employed molecular dynamic (MD) simulations to investigate the binding of RGD peptides to the αvβ3 integrin receptor, a critical target for drug delivery in cancer. Over 100 ns simulations of two RGD peptides revealed spontaneous binding and diffusional processes, mirroring the experimental observation of RGD peptide conjugates specifically targeting integrin-expressing tumor cells. The RGD peptide was selected due to its compact size, specificity, and the αvβ3 integrin’s significant involvement in tumor angiogenesis, proliferation, and metastasis. These computational efforts aim to model the transport of boron atoms via RGD peptides for targeted boron neutron capture therapy (BNCT), a novel strategy for anti-cancer therapeutic delivery. Peptide-binding receptors are vital in cancer therapy, offering specific and selective targets for drug delivery, enabling the selective targeting of cancer cells while sparing healthy tissue. The MD results may shed light on possible delivery routes for specific boron(s) to tumor(s). In particular, the computer-designed RGD-2-peptide + boron(s) and receptor + RGD-1-peptide that MD modeled above fully correspond to their structural behavior in vivo. This statement means that for the computer-engineered RGD-2-peptide + boron(s) and αvβ3 integrin receptor + RGD-1 peptide, all the initial structural data (for RGD peptides and receptor) were completely obtained experimentally using X-rays and are available from the PDB (Protein Data Base); these files fully correspond to the structural forms in vivo. We have modeled the phase transition from a crystalline state to a liquid one; the structural behavior of the modeled multiple RGD-2-peptide, receptor, and RGD-1-peptide systems in an aqueous medium now corresponds to their more adequate physiological liquid state. This work extends previous computational MD studies of “vector–receptor” interactions, specifically focusing on the RGD-αvβ3 integrin pair to elucidate mechanisms for targeted boron delivery to tumors, considering both single and multi-particle boron delivery scenarios.

In conclusion, although the interaction of RGD peptide with the αvβ3 receptor is a well-known paradigm of targeted delivery, as far as the novelty is concerned, there are few works both on the experimental and modeling aspects regarding the mechanism of RGD peptide binding to the αvβ3 integrin receptor, as well as boron transport. In this work, we mainly focused on the atomic/molecular scale events related to the mechanism of RGD peptide binding to the avβ3 integrin receptor. It should be noted that there is currently not much experimental data on the above mechanism, as well as little work being reported on the modeling aspect of the binding of RGD peptides to the avb3 integrin receptor. In this context, the atomic–molecular events related to the RGD peptide and receptor interaction mechanism, perhaps, were modeled as in the experimental setup, where both situations were generated and analyzed: an integrin receptor without RGD (an integrin without RGD), as well as an integrin associated with RGD-1 (an integrin associated with RGD, as in the original PDB file). We used the experimental X-ray structure of the PDB with a standard minimization and relaxation procedure to accurately determine the flexibility of the receptor and its conformational states, thereby performing multiple runs for each MD model (in the range of 10–20 ns initial energy relaxation, up to 50–100 ns large-scale equilibration stages). The MD results illustrate the accumulation of the RGD-2 peptide in a specific region of the integrin, suggesting the existence of multiple binding sites and mechanisms of interaction between the vector and the receptor. Nevertheless, the MD results clearly demonstrate one of the most likely binding mechanisms between RGD-2 and the receptor surface, regardless of whether the αvβ3 integrin receptor with or without RGD-1 was used. In the case of an integrin without RGD-1, RGD-2 + boron(s) would likely find additional sites for incorporation into the receptor. Binding residues of αvβ3 integrin to RGD-2 + boron(s) coincide with the binding of αvβ3 integrin to RGD-1, which is naturally embedded in the receptor. Thus, of the two RGD peptides that were modeled, RGD-1 remains embedded in the protein pocket for the entire simulation period of 50–100 nanoseconds, and animations and distance distributions illustrate the specific, natural binding of RGD-2.

## Figures and Tables

**Table 1:** RGD–αvβ3 integrin pair.

Vector	Receptor
RGD (peptide containing amino acid sequence: L-arginine, glycine, L-aspartic acid)	Integrin αvβ3

**Table 2:** Pharmacological pairs under study.

Vector	Receptor
RGD (peptide containing amino acid sequence: L-arginine, glycine, L-aspartic acid)	Integrin αvβ3
Folic acid	Folate receptors FR-α (tumor marker) FR-β
Boron phenylalanine	Amino acid transporters: • LAT1 (predominantly) • LAT2 • ATB (0,+)

## Data Availability

All data can be found via the links listed in the paper. The original contributions presented in this study are included in the article and Supplementary Materials. Further inquiries can be directed to the corresponding author.

## Supplementary Materials

The following supporting information can be downloaded at: https://www.mdpi.com/article/10.3390/cimb48040411/s1.
