# Supplementary material for: MD Simulation of Vector–Receptor Pharmacologic Pairs for Tumor-Specific Drug Delivery: Transfer of Boron Atoms by RGD Peptide to αvβ3 Integrin Receptor
Source: Curr Issues Mol Biol. 2026 Apr 16;48(4):411. doi: 10.3390/cimb48040411 (PMC13115394; doi:10.3390/cimb48040411)
Supplement: Supplementary file 1 [file cimb-48-00411-s001.zip › cimb-4231597-supplementary.pdf]

Supplementary Materials

Movies Anim_S1, Anim_S2: The animated movies generated by the VMD software on long 100-ns virtual experiments (RGD-2-peptide + receptor avß3-integrin). The relaxed structures in the MD calculations are shown for RGD-2-peptide + boron(s) and receptor αvβ3 -integrin demonstrating the RGD-2 binding mechanism into the receptor in the water environment.

Table S1. The equilibration stage for RGD + receptor interaction dynamics in explicit solvent.

model.explicit.mdin
The Equilibration Stage in Explicit Solvent
&cntrl
imin=0, ! Molecular dynamics and Equilibration (=1, Minimization)
ntc=2, ! SHAKE constraints (=2, hydrogen bond lengths constrain.)
ntf=2, ! Force evaluation (=2, hydrogen bond interact.s omitted)
ntb=1, ! Boundaries (=1, constant volume)
cut=9.0, ! Cutoff
dt=0.002, ! The time step in picoseconds
nstlim=50000000, ! Number of MD steps performed
ig=-1, ! Random seed (=-1, get a number from date and time)
ntwr=10000, ! Restart file written every ntwr steps
ntwx=10000, ! Trajectory file written every ntwx steps
ntpr=10000, ! The mdout and mdinfo files written every ntpr steps
ioutfm=1, ! Trajectory file format (=1, Binary NetCDF)
iwrap=1, ! Translate water molecules into original simulation box
igb=0, ! GB model (=0, explicit solvent)
&end
